# Phase-lag return mappings for a 3 cell multifunctional central pattern generator

**DOI:** 10.1186/1471-2202-13-S1-P188

**Published:** 2012-07-16

**Authors:** Jeremy Wojcik, Robert Clewley, Andrey Shilnikov

**Affiliations:** 1 Department of Mathematics and Statistics, Georgia State University, Atlanta, GA 30033, USA; 2 Neuroscience Institute, Georgia State University, Atlanta, GA 30033, USA

## 

We describe and expand on a novel computational approach to reduce detailed models of central pattern generation to equationless return mapping for the phase lags between the constituting bursting interneurons [1].

Such mappings are then studied geometrically as the model parameters, including coupling properties of inhibitory and excitatory synapses, or external inputs are varied. Bifurcations of the fixed points and invariant circles of the mappings corresponding to various types of rhythmic activity are examined. These changes uncover possible biophysical mechanisms for control and modulation of motor-pattern generation. Our analysis does not require knowledge of the equations that model the system, and so provides a powerful new approach to studying detailed models, applicable to a variety of biological phenomena beyond motor control.

Motifs of three coupled cells are a common network configuration including models of biological central pattern generators. We demonstrate our technique on a motif of three reciprocally coupled, inhibitory and excitatory, cells that is able to produce multiple patterns of bursting rhythms. In particular, we examine the qualitative geometric structure of two-dimensional maps for phase lag between the cells. This reveals the organizing centers of emergent polyrhythmic patterns and their bifurcations, as the asymmetry of the synaptic coupling is varied. The presence of multistability and the types of attractors in the network are shown to be determined by the duty cycle of bursting, as well as coupling interactions.

**Figure 1:**
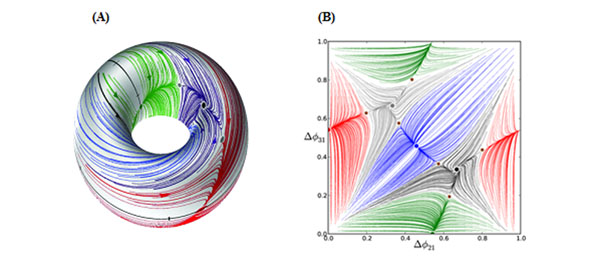
(A). We defined phase-lags for a 3 cell CPG model on a 2D torus. (B) A “flattened” phase-lag mapping showing convergence to 5 distinct attractors. Each stable fixed point represents a rhythmic output of the CPG
